# Artificial intelligence and wearables in sport: performance, injury risk, and wellbeing

**DOI:** 10.3389/frai.2026.1838507

**Published:** 2026-05-29

**Authors:** Walaa Jumah Alkasasbeh, Adam Tawfiq Amawi, Gerasimos V. Grivas, Bekir Erhan Orhan, Thekra Alawamleh

**Affiliations:** 1 Department of Physical Education, School of Sports Sciences, The University of Jordan, Amman, Jordan; 2 Department of Movement Sciences and Sports Training, School of Sport Science, The University of Jordan, Amman, Jordan; 3 Physical Education and Sports, Division of Humanities and Political Sciences, Hellenic Naval Academy, Piraeus, Greece; 4 Faculty of Sports Sciences, Istanbul Aydin University, Istanbul, Türkiye

**Keywords:** artificial intelligence, athlete monitoring, athlete wellbeing, injury prevention, performance optimization, wearable technology

## Abstract

The integration of artificial intelligence (AI) and wearable technologies has reshaped contemporary sport practice by enabling continuous, multidimensional athlete monitoring. Wearable systems generate high-frequency physiological, biomechanical, and behavioral data; however, meaningful interpretation of these datasets requires advanced analytical approaches. This mini review synthesizes current evidence on the combined application of AI and wearable technologies in sport, with emphasis on performance optimisation, injury risk estimation, return-to-play decision support, and athlete wellbeing. The literature indicates that AI-driven models can enhance individualized training prescription, improve workload regulation, and support early identification of maladaptive patterns. Nevertheless, predictive accuracy and practical utility remain highly dependent on data quality, model validation, contextual interpretation, and practitioner expertise. Ethical considerations, including data privacy, algorithm transparency, and responsible governance, represent additional challenges for widespread implementation. Overall, the findings support a human-in-the-loop framework in which AI functions as an advanced decision-support tool rather than an autonomous authority. When applied within structured and context-aware practice models, AI-integrated wearable systems may contribute to more adaptive, individualized, and sustainable athlete management strategies.

## 1 Introduction

Modern sports science has undergone substantial transformation driven by the rapid advancement of digital technologies and data analytics (Rein and Memmert, 2016). Artificial intelligence (AI) and wearable technologies have emerged as key tools for athlete monitoring, performance management, and health-related decision-making in both elite and sub-elite sport settings (Bittencourt et al., 2016). Contemporary wearable systems, including global positioning systems (GPS) (Cummins et al., 2013), inertial measurement units (IMUs) (Camomilla et al., 2018), heart rate and heart rate variability (HRV) monitors (Plews et al., 2013), and sleep-tracking sensors (de Zambotti et al., 2024), enable continuous, non-invasive collection of multidimensional physiological, biomechanical, and behavioral data. This technological evolution has contributed to a shift from experience-based coaching toward data-driven and evidence-informed practice within sport performance environments (AlKasasbeh and Amawi, 2024; Alawamleh and AlKasasbeh, 2024; Marinho and Neiva, 2018; Claudino et al., 2019). Although wearable technologies facilitate large-scale real-time data acquisition, the increasing complexity and volume of these datasets require advanced analytical approaches for meaningful interpretation (Cardinale and Varley, 2017; Marinho and Neiva, 2018). In this context, AI particularly machine learning and deep learning techniques, has become increasingly important for identifying hidden patterns within multidimensional sport data, supporting individualized training prescription, forecasting performance readiness and fatigue, and optimizing training load management (Bittencourt et al., 2016; Marinho and Neiva, 2018; Rossi et al., 2018; Claudino et al., 2019; Rothschild et al., 2024). These developments represent a transition from traditional population-based training models toward adaptive, athlete-specific monitoring and decision-support systems (Buchheit and Simpson, 2017; Foster et al., 2017; Rothschild et al., 2024).

Beyond performance optimization, the integration of AI and wearable technologies has attracted considerable attention in relation to injury risk assessment and return-to-play decision-making (Bittencourt et al., 2016; Rossi et al., 2018; Claudino et al., 2019). Sports injuries, particularly non-contact and overuse conditions, remain a major concern due to their impact on athlete availability, long-term health, and organizational costs (Lutter et al., 2022). Wearable-derived indicators, such as neuromuscular fatigue (Claudino et al., 2019), movement asymmetries, and internal-external workload interactions (Gabbett, 2016), have been incorporated into AI-driven predictive frameworks aimed at detecting early warning signs of injury risk and informing return-to-play decisions (Foster et al., 2017; Rossi et al., 2018). Nevertheless, the reliability, validity, and generalizability of these predictive systems across sports, competitive levels, and demographic groups remain under active investigation.

At the same time, increasing recognition of athlete wellbeing and mental health has broadened the scope of performance monitoring beyond purely physiological parameters (Reardon et al., 2019). Competitive athletes frequently report psychological stress, sleep disturbance, and burnout, highlighting the need for integrated monitoring approaches (Frost et al., 2023; Glandorf et al., 2025). Wearable-derived measures, including HRV (Plews et al., 2013), sleep quality (de Zambotti et al., 2024), and recovery indices (Bestwick-Stevenson et al., 2022), provide indirect indicators of psychophysiological status, while AI-based analytical models offer potential for integrating these signals into comprehensive wellbeing profiles (Claudino et al., 2019; Molavian et al., 2023). However, interpretation of such indicators remains context-dependent and requires integration with subjective assessments and professional judgment.

Despite rapid growth in this research area, performance optimization, injury prevention, and athlete wellbeing are often examined separately within the existing literature (Yung et al., 2022; Forelli et al., 2024). Few reviews have comprehensively explored how AI and wearable technologies jointly support these interrelated domains within a unified conceptual perspective. In addition, important challenges including algorithmic transparency, data privacy, ethical governance, and the risk of over-reliance on automated decision-making, remain insufficiently synthesized (Qi et al., 2024; Habelalmateen and Kumar, 2025).

Therefore, the aim of this Mini review is to critically evaluate current evidence regarding the integration of AI and wearable technologies in sport, with particular emphasis on implications for performance optimization, injury prevention, and athlete wellbeing. By synthesizing multidisciplinary findings, this review seeks to clarify the potential benefits, methodological limitations, and future research directions associated with AI-driven athlete monitoring and decision-support systems.

## 2 Methodology

### 2.1 Study design

This study employed a structured Mini review methodology to critically analyse and synthesise the existing literature on the integration of AI and wearable technologies in sport. The review focused on three interrelated domains: performance optimisation, injury prevention, and athlete wellbeing. A narrative approach was selected due to the multidisciplinary nature of the topic and the substantial heterogeneity observed in study designs, athlete populations, wearable devices, and analytical techniques, which limits the feasibility of formal systematic or meta-analytic procedures. To enhance methodological transparency and rigor, the review was conducted in line with established recommendations for high-quality Mini reviews, including clearly defined search procedures, eligibility criteria, and thematic synthesis.

### 2.2 Literature search strategy

A comprehensive literature search was conducted across major scientific databases commonly used in sport and health sciences, including PubMed, Scopus, Web of Science, and Google Scholar. The search covered publications from January 2010 to March 2026, reflecting the period during which AI applications and wearable technologies have rapidly expanded within sport environments. The search strategy combined relevant keywords and Boolean operators related to artificial intelligence, machine learning, deep learning, wearable technology, athlete monitoring, training load, injury risk prediction, heart rate variability, sleep monitoring, recovery, and athlete wellbeing. In addition to electronic database searches, the reference lists of key review articles, consensus statements, and highly cited empirical studies were manually screened to identify additional relevant publications. The initial search yielded a broad pool of potentially relevant articles, which were subsequently screened according to predefined eligibility criteria.

### 2.3 Study selection process

Following the database searches, a total of 214 records were initially identified through PubMed, Scopus, Web of Science, and Google Scholar. After removal of duplicate records, 168 articles remained for title and abstract screening. Studies were excluded if they were not directly relevant to the objectives of the review, focused primarily on non-sport clinical contexts, lacked discussion of artificial intelligence or wearable technology applications in sport, or did not provide a meaningful conceptual or applied contribution to the topic of the review. Subsequently, the full texts of 67 articles were assessed for eligibility. Following the application of the predefined inclusion and exclusion criteria, 57 studies were ultimately included in the final thematic synthesis of this mini review. Although this review adopted a structured search and screening approach to enhance methodological transparency, it was conducted as a narrative mini review.

### 2.4 Eligibility criteria

Studies were included if they met the following criteria: (1) peer-reviewed articles published in English; (2) studies involving athletic or physically active populations; (3) research examining the use of AI, machine learning, or advanced analytical methods in conjunction with wearable-derived data; and (4) studies addressing at least one of the following outcomes: performance enhancement, injury risk assessment, return-to-play decision support, or athlete wellbeing. Studies were excluded if they focused exclusively on non-athletic clinical populations, lacked a clear application of AI or wearable technologies in sport, or were not sufficiently relevant to the conceptual scope of the review.

### 2.5 Data extraction and synthesis

Relevant information was extracted from each eligible study, including study objectives, athlete characteristics, type of wearable devices, analytical or AI-based methods applied, monitored variables, and principal findings. Rather than performing quantitative aggregation, findings were synthesised thematically and organised into conceptual categories aligned with the objectives of the review. This approach enabled the identification of consistent evidence patterns, emerging technological applications, and methodological limitations across diverse sport contexts, while maintaining a critical and integrative perspective. A summary of the main characteristics and findings of the included studies is presented in Table 1.

**Table 1:** Summary of selected studies included in the mini review.

Study	Sport/population	AI/wearable technology	Main focus	Key findings
Claudino et al. (2019)	Team sports athletes	AI and machine learning models	Injury risk assessment and performance prediction	AI may assist individualized monitoring and injury prediction, but model validity varies across contexts
Rossi et al. (2018)	Soccer players	GPS data + machine learning	Injury forecasting	GPS-derived workload variables improved injury prediction accuracy
Rothschild et al. (2024)	Endurance athletes	Machine learning recovery models	Recovery monitoring	AI-based models supported individualized recovery estimation
Plews et al. (2013)	Elite endurance athletes	HRV monitoring	Training adaptation and recovery	HRV monitoring may assist fatigue and recovery assessment
Seshadri et al. (2020)	Athletes	Wearable technology and analytics	Workload management and injury prevention	Wearable analytics may support workload optimization and reduce injury burden
Wang et al. (2026)	Sports rehabilitation settings	Multimodal AI systems	Injury prediction and rehabilitation	AI integration may improve rehabilitation monitoring and return-to-play decisions
Badicu and Silva (2025)	Athletes	Machine learning approaches	Mental state monitoring	AI-based systems may support psychophysiological monitoring
de Zambotti et al. (2024)	Sleep and circadian research participants	Wearable sleep technologies	Sleep monitoring	Wearables provide useful sleep-related data but require cautious interpretation
Qi et al. (2024)	Sport systems	Digital technologies and AI	Ethics and athlete wellbeing	Highlighted concerns related to privacy, governance, and athlete data protection
Mateus et al. (2025)	Sports science practice	Artificial intelligence systems	Training and health management	AI may enhance decision-support capacity in sports science settings

### 2.6 Ethical and methodological considerations

Given the increasing implementation of AI-based systems in sport practice, particular attention was paid to ethical and methodological considerations, including data privacy, athlete data ownership, algorithmic transparency, interpretability of predictive models, and the risk of over-reliance on automated decision-making. The review adopts a human-in-the-loop perspective, emphasising that AI-generated outputs should function as decision-support tools that complement professional expertise rather than replace contextual judgement. This approach is essential to ensure athlete safety, responsible governance, and sustainable integration of digital technologies in applied sport settings.

In addition, practical ethical considerations surrounding AI-integrated wearable systems require careful attention within sport environments. Athletes should provide informed consent regarding the collection, storage, and analysis of wearable-derived data, with clear communication concerning how these data will be used and who may have access to them. Questions related to athlete data ownership and long-term data storage remain important concerns, particularly when third-party technology providers are involved. Furthermore, insufficient data protection measures may increase the risk of privacy breaches, unauthorized access, or misuse of sensitive physiological and psychological information. Ethical implementation therefore requires transparent governance frameworks, secure data management procedures, and responsible oversight to ensure that AI-based monitoring systems support athlete welfare without compromising autonomy, confidentiality, or professional judgement.

## 3 Theoretical framework

This Mini review is grounded in an integrated theoretical perspective that combines load–response models, the biopsychosocial framework of athlete health, complex systems theory, and data-driven decision-making. Considered collectively, these perspectives provide a conceptual foundation for understanding how wearable technologies and artificial intelligence (AI) influence athlete performance, injury prevention, and wellbeing within dynamic sport environments. Rather than operating as independent constructs, these theoretical models interact to explain how data acquisition, physiological adaptation, psychological regulation, and risk dynamics are interconnected.

### 3.1 Data-driven decision-making in sports

The increasing adoption of wearable technologies has accelerated the transition from experience-based coaching toward data-informed and evidence-based performance management (Migliaccio et al., 2024). High-frequency, multidimensional datasets generated in training and competition require analytical approaches capable of identifying patterns within complex and time-sensitive information streams. AI techniques, particularly machine learning, enable the transformation of large-scale wearable-derived data into actionable insights that support performance tracking, workload regulation, and risk estimation (Dudek et al., 2025; Grivas and Safari, 2025).

However, data-driven decision-making does not replace theoretical principles of training and adaptation. Instead, AI functions as a decision-support mechanism operating within established physiological and performance models.

### 3.2 Load–response models and injury risk

Load–response theory provides a physiological framework explaining how interactions between internal and external training loads influence adaptation and injury risk (Impellizzeri et al., 2019). Performance enhancement occurs when training stimuli are appropriately aligned with an athlete’s adaptive capacity, whereas maladaptation and injury may emerge when cumulative load exceeds recovery potential (Gabbett, 2016). Wearable technologies enable continuous quantification of training load variables, while AI-based predictive models enhance the capacity to analyse temporal load–response relationships. The integration of these tools facilitates earlier identification of maladaptive patterns and supports more informed return-to-play decisions (Wang et al., 2026). Nevertheless, predictive models must be interpreted cautiously, as load–response interactions are influenced by contextual, sport-specific, and individual factors.

### 3.3 Biopsychosocial model of athlete wellbeing

The biopsychosocial model recognises that athletic performance and health are shaped by interrelated biological, psychological, and environmental factors (John et al., 2020). Physiological adaptation alone does not determine performance sustainability; psychological stress, sleep quality, recovery status, and social context also play essential roles in athlete readiness and long-term wellbeing (Alkasasbeh et al., 2026; Charest and Grandner, 2020). Wearable technologies provide indirect markers of psychophysiological status, including heart rate variability (Lehrer and Gevirtz, 2014; Shaffer and Ginsberg, 2017), sleep metrics (Halson, 2014), and recovery-related indicators. AI-based systems facilitate integration of these multidimensional signals into broader monitoring profiles. However, wearable-derived measures of psychological strain remain indirect proxies and should be interpreted alongside subjective reports and clinical expertise to avoid oversimplification of complex mental states.

### 3.4 Complex systems and holistic athlete monitoring

Complex systems theory conceptualises athletes as adaptive systems characterised by non-linear interactions among physiological load, psychological state, environmental stressors, and injury risk (Meeuwisse et al., 2007). Within this framework, injuries and performance fluctuations emerge from dynamic and recursive interactions rather than isolated risk factors (Meeuwisse et al., 2007; Davids et al., 2013). AI-driven analytics are particularly suited to modelling such high-dimensional and time-dependent interactions, as machine learning approaches can detect patterns not easily captured by traditional linear methods. At the same time, the application of AI within complex sport systems raises ethical and governance considerations, including transparency, interpretability, and responsible data use (Harlow, 2018; Zadeh et al., 2021). These concerns reinforce the importance of maintaining human oversight in AI-assisted athlete monitoring frameworks.

## 4 Applications of AI and wearable technologies in sports practice

The integration of AI with wearable technologies has substantially reshaped contemporary sport practice by enabling continuous, individualized, and context-aware athlete monitoring (Claudino et al., 2019). Rather than relying on isolated performance indicators, modern AI-driven systems synthesize large volumes of physiological, biomechanical, and behavioral data into structured decision-support outputs that inform training design, competition preparation, and recovery management (Buchheit and Simpson, 2017; Cardinale and Varley, 2017; Claudino et al., 2019). This integration reflects a broader shift toward multidimensional athlete regulation frameworks, in which data streams are interpreted within performance and health models rather than as independent metrics.

### 4.1 Performance optimization and training personalization

Wearable technologies such as GPS units, inertial measurement units (IMUs), heart rate monitors, and sleep sensors generate high-resolution data reflecting both external and internal training loads (Cardinale and Varley, 2017; Sperlich and Holmberg, 2017b; Impellizzeri et al., 2019). AI-based analytical techniques, particularly machine learning approaches, enable the processing of these multidimensional datasets to detect patterns associated with performance adaptation, fatigue accumulation, and recovery status (Bittencourt et al., 2016; Claudino et al., 2019).

This capability supports the development of individualized training prescriptions that extend beyond traditional population-based models toward athlete-specific load management strategies (Gabbett, 2016; Buchheit and Simpson, 2017; Rothschild et al., 2024). In applied settings, AI systems are increasingly employed to estimate short-term performance readiness, identify deviations from expected adaptation trajectories, and assist in dynamic training periodization (Grivas and Safari, 2025). By learning from longitudinal athlete data, these systems can adapt training intensity and volume according to individual responses to comparable workloads (Buchheit and Simpson, 2017; Claudino et al., 2019; Rothschild et al., 2024).

However, the effectiveness of such systems depends heavily on data quality, ecological validity, and appropriate contextual interpretation. AI-driven outputs should therefore be understood as probabilistic guidance rather than deterministic prescriptions, reinforcing their role as decision-support tools that enhance, but do not replace, practitioner expertise (Mateus et al., 2025).

### 4.2 Injury risk assessment and return-to-play decision support

Injury prevention represents one of the most extensively investigated applications of AI and wearable technologies in sport (Bittencourt et al., 2016). Wearable-derived indicators related to cumulative load, neuromuscular fatigue, movement asymmetries, and workload variability have been integrated into predictive models aimed at estimating injury risk, particularly for non-contact and overuse injuries (Seshadri et al., 2020; Zadeh et al., 2021). These models frequently rely on time-series analyses to detect subtle shifts in load–response relationships that may precede injury onset (Meeuwisse et al., 2007; Rossi et al., 2018).

Within return-to-play contexts, AI-assisted monitoring systems support clinicians and performance staff by tracking recovery trajectories and comparing post-injury metrics with pre-injury baselines (Claudino et al., 2019; Impellizzeri et al., 2019; Wang et al., 2026). Such comparisons may facilitate more objective progression decisions and improve alignment between rehabilitation stages and competitive demands (Grivas and Safari, 2025).

Nevertheless, predictive accuracy remains variable across sports, populations, and competitive levels (Kucera et al., 2005; Thorpe et al., 2015; Hulin et al., 2016; Van Eetvelde et al., 2021). This variability underscores the importance of contextual expertise and highlights the risk of over-reliance on algorithmic predictions. Injury risk is inherently multifactorial and dynamic, and predictive outputs should be interpreted within broader clinical and performance frameworks.

### 4.3 Athlete wellbeing and psychophysiological monitoring

Beyond performance and injury management, AI-integrated wearable systems are increasingly applied to monitor athlete wellbeing and mental health (Badicu and Silva, 2025). Indicators such as heart rate variability, sleep quality, recovery indices, and daily load fluctuations provide indirect markers of psychological stress, fatigue, and overall readiness (Plews et al., 2013; Halson, 2014; Charest and Grandner, 2020). AI-based frameworks enable the integration of these variables into more comprehensive wellbeing profiles that may support early identification of maladaptive responses such as burnout, chronic fatigue, or excessive stress (Alkasasbeh et al., 2026; Amawi et al., 2026).

In applied environments, these systems contribute to more holistic athlete management strategies by aligning physical training metrics with psychophysiological indicators (Alkasasbeh et al., 2024; McLean et al., 2010; Sperlich and Holmberg, 2017a; Kellmann et al., 2018). However, wearable-derived measures of mental wellbeing remain indirect and context-sensitive (Halson, 2014; Reardon et al., 2019). Consequently, their interpretation requires careful integration with validated psychological assessments, athlete self-reports, and professional judgement to avoid reductionist interpretations of complex psychological states.

## 5 Discussion

The findings of this mini review indicate that the integration of AI with wearable technologies represents a substantial evolution in contemporary sports science practice. Rather than relying exclusively on experiential judgment, practitioners are increasingly supported by data-driven approaches that integrate high-frequency physiological, biomechanical, and behavioral information (Bittencourt et al., 2016; Marinho and Neiva, 2018; Claudino et al., 2019). Wearable systems such as GPS devices, inertial measurement units, and heart rate monitors enable continuous quantification of internal and external load variables, providing a more granular understanding of performance dynamics than traditional assessment methods (Cummins et al., 2013; Camomilla et al., 2018; Impellizzeri et al., 2019).

From a performance optimisation perspective, AI-supported systems appear to facilitate more individualized training prescriptions by accounting for inter-individual variability in adaptation to training loads (Buchheit and Simpson, 2017; Foster et al., 2017; Rothschild et al., 2024). Models trained on longitudinal athlete-specific data may improve estimation of readiness, fatigue, and recovery compared with population-based averages (Rossi et al., 2018). However, these potential advantages must be interpreted cautiously. Model performance is highly dependent on data quality, contextual factors, and sport-specific characteristics. Generalisability across competitive levels and athlete populations remains limited, highlighting a persistent gap between technological capability and consistent real-world application.

In the domain of injury prevention, the literature supports a growing role for AI-driven approaches in estimating risk for non-contact and overuse injuries (Bittencourt et al., 2016; Rossi et al., 2018; Zadeh et al., 2021). Predictive frameworks incorporating cumulative workload, neuromuscular fatigue, and movement asymmetries align with complex systems models of sports injury, which conceptualise injury as the result of dynamic and non-linear interactions among multiple risk factors (Meeuwisse et al., 2007). Nevertheless, predictive accuracy remains inconsistent, and false-positive or false-negative outputs may carry practical consequences. Injury risk is inherently multifactorial and context-dependent, and algorithmic predictions should therefore complement, rather than replace, clinical reasoning and practitioner expertise (Thorpe et al., 2015; Hulin et al., 2016).

AI-assisted monitoring also demonstrates potential within return-to-play decision-making frameworks. By comparing post-injury recovery trajectories with pre-injury baselines, practitioners may obtain additional objective information to guide progression decisions (Impellizzeri et al., 2019; Grivas and Safari, 2025). However, the assumption that algorithmic alignment equates to readiness for competition remains problematic. Successful return-to-play decisions require integration of physiological data, functional testing, sport-specific demands, and psychological readiness. Over-reliance on model outputs may oversimplify these multidimensional considerations.

Beyond performance and injury management, this review highlights the expanding application of AI-integrated wearable systems in monitoring athlete wellbeing and mental health. Indicators such as heart rate variability, sleep quality, and recovery indices provide indirect proxies of psychophysiological status (Plews et al., 2013; Halson, 2014; Charest and Grandner, 2020). When interpreted within a biopsychosocial framework (John et al., 2020), these signals may contribute to earlier identification of maladaptive responses such as chronic fatigue or burnout (Frost et al., 2023; Glandorf et al., 2025). However, wearable-derived metrics represent indirect indicators of psychophysiological and psychological status and should not be interpreted as substitutes for clinical judgment, validated psychological assessment, or structured athlete self-report measures (Reardon et al., 2019). In addition, the effectiveness of AI-based predictive systems remains strongly dependent on dataset size, data quality, and model validation procedures. Many existing studies rely on relatively small or homogeneous athlete samples, which may increase the risk of overfitting and reduce the generalizability of predictive outputs across sports and competitive levels. Furthermore, external validation of AI models remains limited within applied sport environments, making real-world implementation and reproducibility ongoing challenges.

Importantly, the increasing integration of AI into athlete monitoring systems raises ethical and governance considerations. Issues related to data privacy, ownership, algorithmic transparency, and interpretability require structured oversight (Harlow, 2018; Qi et al., 2024). As predictive models become more embedded in training environments, the risk of over-automation and diminished professional autonomy must be acknowledged. The technological sophistication of AI does not eliminate uncertainty inherent in human performance systems.

Overall, the present findings suggest that AI-assisted wearable technologies may support more adaptive, individualized, and context-aware athlete monitoring within contemporary sport environments when implemented responsibly and interpreted within appropriate clinical, ethical, and sport-specific contexts (Claudino et al., 2019; Mateus et al., 2025).

### 5.1 Limitations

Despite the contributions of this mini review, several limitations should be considered when interpreting its findings. First, the review was restricted to peer-reviewed articles published in English, which may have resulted in the exclusion of relevant studies reported in other languages or unpublished technical reports. Given the rapid evolution of artificial intelligence applications and wearable technologies, some emerging evidence may also exist in industry reports or conference proceedings that were not included in the present synthesis. Second, substantial heterogeneity was observed across the included studies in terms of athlete populations, competitive levels, wearable devices, data acquisition protocols, and analytical approaches. Variability in sensor accuracy, data preprocessing procedures, and model validation strategies limits direct comparison between studies and complicates interpretation of reported predictive performance. In particular, differences in how internal and external load variables are operationalized may affect the consistency of findings across sport contexts. Third, much of the existing literature focuses on specific sports, team-based settings, or elite athlete samples. This concentration may restrict the generalizability of conclusions to other populations, including youth athletes, recreational participants, or underrepresented sport disciplines. Additionally, many AI-based models have been developed using relatively small or homogeneous datasets, which may increase the risk of overfitting and reduce external validity. Finally, due to the narrative design of this review, no formal risk-of-bias assessment or quantitative meta-analysis was conducted. Such limitations may also reduce model robustness and limit the applicability of predictive outputs across different athlete populations and real-world sport environments. Findings were synthesised conceptually rather than statistically, and effect sizes were not aggregated. While this approach allows broader theoretical integration across heterogeneous domains, it also limits the capacity to draw definitive conclusions regarding the magnitude of effects or predictive accuracy of specific AI frameworks.

### 5.2 Practical implications for sports practice

The findings of this review indicate that AI-integrated wearable technologies can provide meaningful decision-support within applied sport environments, provided their implementation is structured and context-sensitive. In training settings, continuous monitoring of internal and external load variables enables practitioners to better detect individual response patterns, identify deviations from expected adaptation trajectories, and adjust training volume or intensity accordingly. When longitudinal athlete-specific data are systematically collected and interpreted, AI-assisted systems may enhance the precision of workload management and periodization planning (AlKasasbeh et al., 2025). In injury prevention contexts, predictive models derived from wearable data can contribute to earlier identification of workload spikes, recovery deficits, or maladaptive patterns associated with increased injury risk. However, these indicators should be integrated within broader clinical and performance assessment frameworks, including functional testing and sport-specific evaluation. AI outputs should therefore be interpreted as probabilistic risk estimations rather than deterministic conclusions. Regarding athlete wellbeing, the integration of wearable-derived metrics, such as sleep quality, heart rate variability, and recovery indices with structured self-report tools may strengthen early recognition of excessive fatigue, psychological stress, or potential burnout. Multidimensional monitoring strategies that combine objective and subjective data are likely to provide more accurate representations of athlete readiness than isolated metrics (AlKasasbeh and Amawi, 2023). Importantly, successful implementation of AI-based monitoring requires practitioner education, transparent communication with athletes regarding data use, and clearly defined decision-making protocols. The practical value of these technologies lies not in automation, but in their capacity to enhance situational awareness and support informed professional judgment within complex sport environments.

### 5.3 Future research directions

Future research should prioritise the development of more methodologically rigorous and longitudinal study designs that examine the sustained impact of AI-driven monitoring systems on performance adaptation, injury incidence, and athlete wellbeing. Short-term validation studies, while informative, are insufficient to determine whether algorithm-guided adjustments meaningfully improve long-term training outcomes or reduce injury burden across competitive seasons. There is a need for larger, multi-center investigations that include diverse athletic populations across different sports, competitive levels, and age groups. Expanding datasets beyond elite team-sport environments would enhance the external validity and generalisability of predictive models. In addition, future studies should adopt standardized definitions of internal and external load variables, injury classification, and model performance metrics to facilitate cross-study comparison and reproducibility. From a technological perspective, greater emphasis should be placed on model transparency, explainability, and validation procedures. Black-box predictive systems may limit practitioner trust and impede practical implementation. Research focusing on interpretable machine learning approaches, robust cross-validation strategies, and independent model testing is necessary to strengthen scientific credibility in applied sport contexts. Finally, integrating objective wearable-derived metrics with validated psychological self-report instruments and contextual performance indicators may contribute to more comprehensive, multidimensional models of athlete health. Such integrative approaches could improve understanding of how physiological load, psychological stress, and environmental factors interact dynamically over time within complex sport systems.

## 6 Conclusion

This mini review underscores the expanding role of artificial intelligence and wearable technologies in reshaping contemporary sport practice. By enabling continuous, multidimensional monitoring of physiological, biomechanical, and psychophysiological variables, these systems offer new opportunities for individualized performance optimisation, injury risk estimation, and holistic athlete management. However, technological capability does not automatically translate into improved outcomes. The effectiveness of AI-driven monitoring frameworks remains contingent upon data quality, methodological rigor, contextual interpretation, and practitioner expertise. Predictive models operate within complex and dynamic sport environments, where uncertainty and variability are inherent. The evidence synthesised in this review supports a human-in-the-loop paradigm in which AI functions as an advanced decision-support mechanism rather than an autonomous authority. Responsible implementation requires transparent algorithms, ethical data governance, and integration with established performance and clinical frameworks.

Nevertheless, despite the promising potential of these technologies, current evidence remains limited by methodological heterogeneity, variable model validation procedures, and insufficient real-world implementation studies. Further longitudinal and externally validated research is required to determine the reliability, generalizability, and practical effectiveness of AI-driven monitoring systems before their widespread adoption across diverse sport settings.

AI-assisted wearable technologies may contribute to enhanced regulatory capacity, more individualized athlete management, and more informed sport practice when supported by appropriate validation, ethical oversight, and contextual interpretation.
